# *In vitro* antibacterial activity and mechanism of cell-free supernatant of *Bacillus velezensis* Ea73 against *Streptococcus agalactiae*, a predominant bacteria that causes mastitis in cows

**DOI:** 10.3389/fvets.2025.1682691

**Published:** 2025-11-21

**Authors:** Siqi Peng, Samuel Kumi Okyere, Lin Cheng, Ziyao Tang, Feng Yang, Xi Liu, Yanchun Hu

**Affiliations:** 1 Agricultural Animal Diseases and Veterinary Public Health Key Laboratory of Sichuan Province, College of Veterinary Medicine, Sichuan Agricultural University, Chengdu, China; 2 Institute of Animal Husbandry Sciences of Ganzi Tibetan Autonomous Prefecture, Kangding, Sichuan, China

**Keywords:** *Bacillus velezensis* Ea73, *Streptococcus agalactiae*, *in vitro* antibacterial activity, biofilm formation, cell free supernatant

## Abstract

*Streptococcus agalactiae* (*S. agalactiae* or GBS) is the main pathogen causing subclinical mastitis in dairy cows. This infection has seriously affected the development of the global dairy industry and thus requires urgent mitigation strategies to reduce its spread. Thus endophytes which are potential sources of novel bioactive antibacterial compounds can effectively be harnessed to address the issues of bacterial infections and resistance in animal production. The aim of this study was to investigate the *in vitro* antibacterial activity and potential mechanism of *Bacillus velezensis* Ea73 (an endophyte from *Ageratina adenophora* plant) cell free supernatant (CFS) against *S. agalactiae* isolated from cows. The antibacterial activity of Ea73 CFS against *S. agalactiae* was analyzed by plate and double dilution methods. Biofilm staining and transmission electron microscopy were used to explore the effects of Ea73 CFS on biofilm formation, structure and extracellular polymeric substances in the *S. agalactiae*. In addition, network pharmacology was used to further explore the relevant mechanisms of Ea73 CFS in treating diseases mediated by *S. agalactiae*. The results showed that the inhibition zone diameter of Ea73 CFS against *S. agalactiae* was 12 mm, and the minimum biofilm inhibition concentration was 10 mg/mL. After treatment with Ea73 CFS, the biofilm formation was significantly inhibited, the structure of the generated biofilm was disrupted, and the metabolic activity of bacterial cells was significantly reduced. The results from this study indicated that EA73 CFS significantly inhibits *S. agalactiae* activity via biofilm eradication and hence can serve as a promising antibacterial reagent.

## 1 Introduction

*Streptococcus agalactiae* is one of the common and main pathogenic bacteria that cause cow mastitis. Mastitis is the most common disease of dairy cattle worldwide, causing economic losses due to reduced yield and poor quality of milk (1). Over the years, several researchers have reported the prevalence of mastitis, both clinical and subclinical, is as high as 81.1% with Staphylococcus and Streptococcus being the dominant pathogenic species (1). The virulence factors of *S. agalactiae* have the function of adhesion and invasion of body cells, causing the bacteria to form a biofilm on the surface of cow breasts, thereby interfering with the normal immune function of the body, causing subclinical bovine mastitis and recurrent infections (2, 3, 52). In addition, *S. agalactiae* also causes neonatal pneumonia, septic shock syndrome, high mortality meningitis, and various diseases in other animals (4). Therefore, the development of effective mitigation strategies for controlling and preventing *S. agalactiae* mastitis is of great significance for the economic efficiency and public health safety of breeding farms.

Antibiotic treatment has been an effective treatment for bovine mastitis, especially in the treatment of acute, latent, and multiple mastitis, as well as in the quality control of dairy products (5). However, due to the arbitrary use of antibiotics, the number of drug-resistant strains has gradually increased, and bacteria have become increasingly resistant to antibiotics. This poses significant challenges to the treatment of mastitis in dairy cows. Therefore, there is an urgent need to identify new antimicrobial agents with high antimicrobial activity and low drug resistance.

*Ageratina adenophora* (*A. adenophora*) is an invasive poisonous weed widely distributed across the world. This plant has been reported to cause great harm to my global agricultural production and development (6–8). However, in recent years the research focus of this plant has been shifted from toxicity study to biological utilization of the plants endophytes resources for the production of new bioactive compounds (9–11). Researchers have found that *A. adenophora* extract has insecticidal, antibacterial, antitumor, antiviral, and antioxidant properties, which are related to its endophytic fungi (12–15). Our previous studies similarly reported antibacterial activities of extracts from both bacteria and fungi endophytes that were isolated from *A. adenophora* (8, 10). However, these studies were mainly focused on *Staphylococcus aureus* and *Escherichia coli*; thus, there is the need to investigate the antibacterial activity and mechanisms associated with these extracts in other strains of pathogenic bacteria to validate its effects.

*Bacillus velezensis* EA73, an endophyte from *Ageratina adenophora*, has shown excellent antibacterial activity against *S. aureus* and *E. coli* (9, 16). However, its activity on other strains of pathogenic bacteria such as *S. agalactiae* as well as the molecular mechanism involved in the endophytic bacteria’s antibacterial properties has not been fully elucidated yet. Therefore, in this study, we focused on investigating the antibacterial activity and molecular mechanisms of *B. velezensis* EA73 CFS against *S. agalactiae* using *in vitro* methods and network pharmacology. This study will provide information on how *B. velezensis* EA73 CFS exhibits its antimicrobial activity to help us develop novel drugs for the treatment of mastitis.

## 2 Materials and methods

### 2.1 Strain and growth conditions of *Bacillus velezensis* EA73 and *Streptococcus agalactiae* (GBS)

*Bacillus velezensis* EA73 (GenBank no. MZ540895) was obtained from Hu Lab, Sichuan Agricultural University, and after routine passaging culture, it was preserved at −80 °C in a Luria–Bertani medium with 20% glycerol (16). *Streptococcus agalactiae* (standard strain MK330595) was presented as a lyophilized powder from Zuo Lab, Sichuan Agricultural University; activated and preserved in BHI broth medium with sterile 60% glycerol at a ratio of 1:1 at −80 °C.

### 2.2 Preparation of EA73 CFS

The Ea73 activation solution was inoculated into a fermentation medium (yeast extract: 6.55 g/L; peptone: 6.61 g/L; NaCl: 20.00 g/L), and fermentation was carried out in a 180 r/min shaker according to fermentation parameters (initial pH: 7.95; temperature: 27.97 °C; time: 51.04 h) (16). The fermentation broth was extracted several times with ethyl acetate at a ratio of 3:1, and the organic phase was collected and concentrated with a rotary evaporator at 45 °C under reduced pressure to obtain the concentrate, which was decontaminated with a 0.22 μm microporous membrane filter and then freeze-dried to obtain the dry substance (Freeze-dryer FDR2110, Rikaku eyela, Tokyo, Japan). The dry substance was stored at −20 °C until use (16).

### 2.3 Antibacterial activity assay

The bacterial suspension was centrifuged at 4000 r/min for 10 min, the bacterial precipitate was collected, and the suspension was suspended in sterile saline to 10^6^ CFU/mL, which was the pathogen suspension. Using the punching method (9), under sterile conditions, 100 μL of the bacterial suspension was aspirated and spread on blood and ordinary agar plates. After drying, it was punched with a 9 mm diameter puncher. 60 μL of Ea73 CFS was added to each well. Sterile normal saline was used as a blank control. After the culture medium was placed in a 37 °C incubator for 24 h, the diameter of the inhibition zone was measured with a vernier caliper using the cross-cross method to evaluate its antibacterial activity. The antibacterial experiment was repeated three times and the average value was taken.

### 2.4 Minimum inhibitory concentration determination

The antibacterial assay was performed using a 96-well plate according to the micro-dilution method. The antibacterial assay consisted of three parallel experimental groups, one blank control group, a positive control, and a negative control. 100 μL of BHI culture medium was added to all microwells in advance. Experimental group: 174.4 μL of Ea73 CFS at a concentration of 0.02 g/mL (i.e., a 200 μL system) was added to the first column of microwells. After thorough mixing with 25.6 μL of pre-added *S. agalactiae* bacterial solution, 100 μL of the mixture was pipetted into the second column. After thorough mixing, 100 μL of the mixture was pipetted into the third column. The mixture was serially diluted to the last column. For the blank control, 100 μL each of BHI broth and normal saline was added. For the negative control, 100 μL each of Ea73 CFS at a concentration of 0.02 g/mL and BHI broth was added. For the positive control, 100 μL each of bacterial solution and BHI broth was added. Finally, the microplate was incubated at 37 °C for 24 h. After incubation, the microplate was placed in a microplate reader and the absorbance was read at 560 nm. The MIC of Ea73 against *S. agalactiae* was determined based on the absorbance.

### 2.5 Determination of MBIC

The MBIC of EA73 CFS against *S. agalactiae* was assessed using crystal violet staining and CFU counting (17). A total of 200 μL of sterile TBS and a 1% (v/v) *S. agalactiae* bacterial suspension (10^8^ CFU/mL) was added to 96-well plates. The plates were incubated at 37 °C for 48 h to form a biofilm, and the supernatant was discarded. The plates were subsequently washed three times with PBS. A gradient concentration of EA73 CFS (0.16–10.24 × 10–3 g/mL) was added, and the mixture was incubated at 37 °C for 24 h. The culture medium was discarded, and the planktonic bacteria were rinsed with sterile PBS and discarded. Then, sterile PBS was added again, and the biofilm was suspended by repeated blowing. The suspension was diluted and spread evenly on a BHI plate for CFU counting. The results were observed after 24 h of incubation at 37 °C. The experiment was repeated three times, and the average value was taken.

After incubation of the biofilm and CFS treatment, the planktonic bacteria were rinsed with sterile PBS, and the biofilm was stained with 0.1% crystal violet for 15 min. After, it was rinsed with sterile PBS and dissolved with 95% ethanol. The OD value at 595 nm of the solution in the culture wells was determined via a multifunctional fluorescence chemiluminescence immunometer.

### 2.6 Antibiotic sensitivity test

Antibiotic sensitivity patterns were evaluated using the method from Okyere et al. (53). 100 μL of *S. agalactiae* suspension was taken with a pipette and placed in a blood plate. The bacterial solution was evenly spread on the plate with a sterilized glass bead. The plate was covered and left to stand for about 15 min until the surface of the plate was slightly dry. Sterilized tweezers were used to stick the antibiotic disks on the surface of the culture medium by gentle pressing. Three antibiotic disks were stuck on each plate. Finally, the plate was placed in a 37 °C constant temperature biochemical incubator for 24 h. The diameter of the inhibition zone was measured with a vernier caliper, and the sensitivity of the strain to antibiotics was determined. The selected antimicrobial drugs were: penicillin, gentamicin, vancomycin, ofloxacin, ampicillin, novobiocin, chloramphenicol, cefoperazone, enrofloxacin, clindamycin, and erythromycin. Disks immersed in sterile water were used as the negative controls.

### 2.7 *S. agalactiae* biofilm-forming ability assay

100 μL of sterile BHI and a 1% (v/v) *S. agalactiae* bacterial suspension (10^8^ CFU/mL, the bacterial suspension was diluted to an OD600 equal to 0.9) were added to 96-well plates and incubated at 37 °C for 12, 24, 48, 72, 96, and 120 h. After incubation, the culture medium was discarded and the plates were gently washed with PBS to remove floating bacteria. The plates were then fixed with 100 μL of methanol solution at room temperature for 15 min, after which the methanol was discarded and the plates were air-dried in a biosafety cabinet. The plates were stained with 100 μL of 0.1% crystal violet solution at room temperature for 5 min. Excess dye was removed with sterile PBS and air-dried on an ultraclean bench. 100 μL of 33% glacial acetic acid was added to the plate and incubated at 37 °C for 30 min. The OD values of the solutions in the wells were measured at a wavelength of 570 nm using a microplate reader. Wells with no bacteria were used as a blank group. The experiment was repeated three times, and the average of the three measurements was used as the final result.

### 2.8 Laser confocal microscopy observation

5 mL of sterile BHI, 1% (v/v) *S. agalactiae* bacterial suspension (10^8^ CFU/mL) and a sterile coverslip (2 cm × 2 cm) were added to a 6-well plate. The plates were incubated at 37 °C for 24 h to form a biofilm. The culture medium was discarded and washed with sterile PBS. Then, Ea73 CFS with a concentration of 1/2 MBIC was added. The wells with the same amount of PBS were used as the control group. After incubation at 37 °C for 4 h, the coverslips were removed and DAPI (10 μg/mL) was added for staining in the dark for 20 min. The biofilm structure was then observed using a confocal laser scanning microscope (CLSM) (18).

### 2.9 Effect of Ea73 CFS on bacterial metabolism in *S. agalactiae* biofilms

The *S. agalactiae* biofilms were cultured on sterile coverslips, as described previously, and then the culture medium was discarded and washed with sterile PBS. Ea73 CFS with concentrations of 1/2 MBIC, MBIC, and 2 MBIC were added to treat the biofilm for 24 h. The control group was treated without Ea73 CFS. The glass slide was removed and placed in sterile water for 15 min to separate the biofilm bacteria from the glass slide. 10% resazurin solution was added to the bacterial suspension, and the suspension was shaken at 37 °C in the dark for 2 h. The supernatant was centrifuged at 10,000 rpm for 10 min at 4 °C. The absorbance at 560 nm excitation wavelength and 593 nm emission wavelength was measured using a microplate reader.

### 2.10 Effects on extracellular polymeric substances (EPS) of *S. agalactiae* biofilms

#### 2.10.1 Enzymatic hydrolysis assay

Biofilms were cultured in 96-well plates as mentioned above and after incubation, the culture medium was discarded. Four groups of samples were set up: one blank control group and the remaining three experimental groups, with 10 replicate wells per group. The blank control group was treated with sterile PBS, while the experimental groups were treated with sodium periodate (10 μM), DNase I (2 mg/mL), and proteinase K (100 μg/mL), respectively. The 96-well plates were incubated at 37 °C for 2 h and stained with crystal violet. Finally, after decolorization with ethanol for 30 min, the OD values at 595 nm were measured using a microplate reader.

#### 2.10.2 Effects of Ea73 CFS on extracellular proteins

Culturing of the biofilms was performed according to the methods described previously, and EA73 CFS (1/2 MBIC, MBIC, or 2 MBIC) was added while the bacteria were being cultured. After the incubation was complete, the culture media was discarded, and the mixture was rinsed with PBS to remove free bacteria from the surface. The cleaned coverslips were sonicated in PBS for 1 h, after which the bacterial suspension was centrifuged at 10,000 rpm for 10 min as an EPS sample for backup. Extracellular proteins were measured by the Coomassie Brilliant Blue method using a protein content assay kit (Biosharp Co., Ltd., Beijing, China). The OD_595nm_ value was measured using a UV spectrophotometer.

#### 2.10.3 Statistical analysis

Experiments were performed in triplicate. Statistical analyses were conducted using GraphPad Prism 8 (GraphPad Inc., La Jolla, CA, USA). The results are shown as the mean ± standard error. Significant differences in the mean values were estimated by one-way analysis of variance (ANOVA) and Student’s t-test.

### 2.11 Ea73 CFS network pharmacology analysis

#### 2.11.1 Target prediction and collection of Ea73 CFS and GBS

Reference was made to the main components of Ea73 CFS obtained by LC–MS/MS by Tang et al. (16). Compounds with a relative content greater than 1% were selected, and the SMILE names of the main components were obtained in Pubchem.^1^ These were then imported into the Swiss target prediction database.^2^ The species was selected as “*homo sapiens*,” and targets with a probability value greater than 0 were screened and used as gene targets. The keyword “*Streptococcus agalactiae*” was entered into the DisGeNET database^3^ and the GeneCards database^4^ and the genes with a relevance score > 5.0 were selected in the GeneCards database. The results obtained from the two databases were summarized, and duplicates were removed to obtain the disease gene targets (19).

#### 2.11.2 Analysis of the disease network mediated by Ea73 CFS main components, targets, and *S. agalactiae*

The interaction between the main active ingredient targets of Ea73 CFS and *S. agalactiae* targets was analyzed using the Venny 2.1.0^5^ website, and the intersection gene targets were obtained. The PPI network diagram was constructed using Cytoscape 3.9.1 software, and the centrality of each node was calculated using the Centiscape 2.2 plug-in (20).

#### 2.11.3 Construction of a target network for EA73 CFS and bovine mastitis (PPI network construction)

Using the STRING^6^ platform, we imported common targets related to the main active ingredient of EA73 CFS and *S. agalactiae*-mediated disease targets. We set the target to “*Streptococcus agalactiae*” and selected the highest confidence level of 0.900. We then hid the free gene nodes and obtained protein–protein interaction relationships.

#### 2.11.4 GO functional annotation and KEGG signaling pathway enrichment analysis

We performed enrichment analysis on shared drug-disease targets in the DAVID database^7^ with “official gene symbol” as the identifier, “*S. agalactiae*” as the species, and used microbial information to generate GO and KEGG enrichment analyses. The top 20 *p*-values were selected as the screening criteria, and the GO and KEGG enrichment analyses were performed by microbial letter mapping.^8^ GO functional analysis included biological process (BP), molecular function (MF), and cellular component (CC). The results were also visualized through the microbiology platform.

#### 2.11.5 Molecular docking computer simulation verification

Molecular docking verification was performed on the main active ingredient and core target protein of the core drug pair using AutoDock Vina 1.1.2. The structures of the core target protein and the top three active ingredients of the effector target were downloaded from the PDB and pubchem websites^9^, respectively. After docking, the structures were visualized using PyMOL 2.6.

## 3 Results

### 3.1 Morphological observation

**Figure 1:**
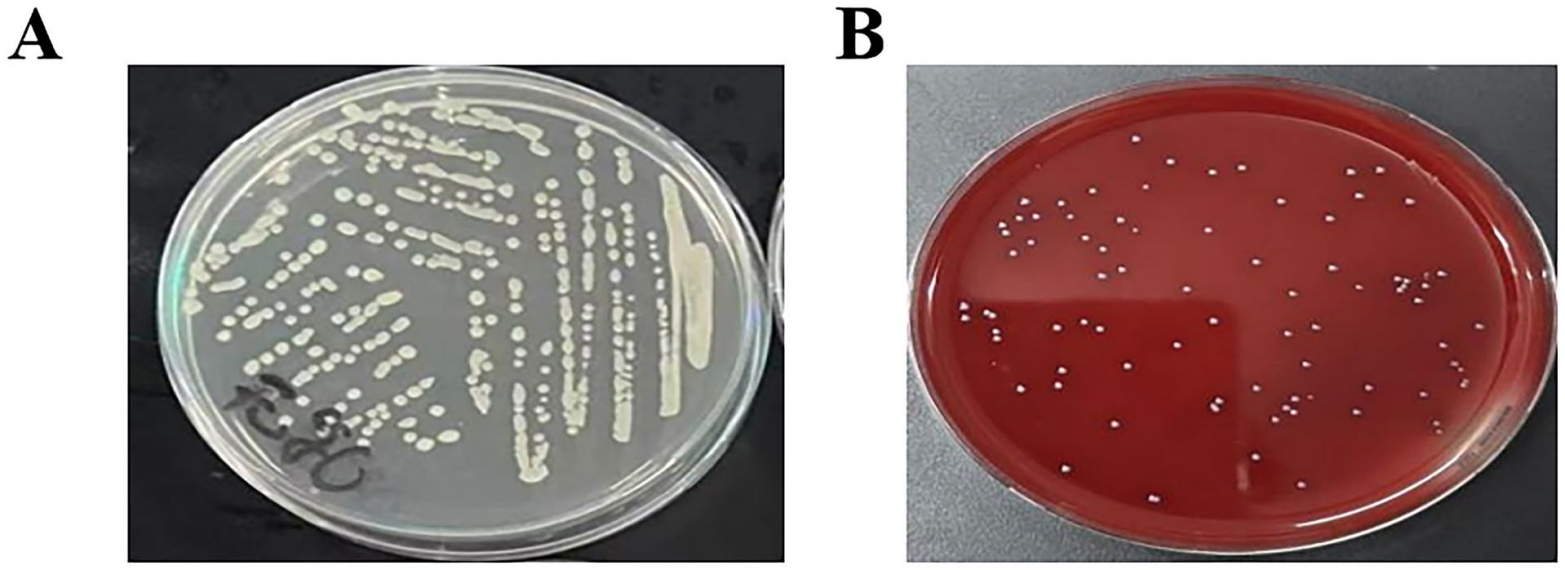
Characteristics of isolated *S. agalactiae* colonies **(A)**. Characteristics of isolated colonies (LB agar plate) **(B)**. Characteristics of isolated colonies (Blood agar plate).

The isolated strain was streaked onto ordinary plates and blood plates, and cultured at 37 °C for 24 h. As shown in Figures 1A,B, the colony morphology was white, round, with neat edges and a smooth and moist surface.

### 3.2 Antibacterial activity assay

The *S. agalactiae* bacterial solution was spread on blood agar plates and ordinary agar plates for comparison. The diameter of the inhibition zone of Ea73 CFS against *S. agalactiae* was measured. The diameter of the inhibition zone of Ea73 CFS against *S. agalactiae* was measured on the blood agar plate was 14 mm, and that on the LB agar plate was 12 mm. Based on the criteria that the inhibition zone diameter ≥ 20 mm is extremely sensitive, 15–20 mm is highly sensitive, 10–15 mm is moderately sensitive, and less than 10 mm is low sensitive, *S. agalactiae* was determined to be moderately sensitive to Ea73 CFS, and hence have a good antibacterial activity.

### 3.3 Antibiotics susceptibility testing

11 Antibiotics were used for the antibiotic susceptibility testing. The results are shown in Table 1. *S. agalactiae* strains exhibited varying susceptibility to the 11 antimicrobial susceptibility tablets. The strains were most sensitive to penicillin, cefoperazone, ampicillin, and clindamycin, while exhibiting lower susceptibility to the commonly used clinical drug gentamicin.

**Table 1:** Criteria for determining the diameter of the inhibition zone in drug sensitivity test.

Antibiotics	Inhibition zone diameter (mm)	Sensitivity
Novobiocin	12	I
Enrofloxacin	15	I
Gentamicin	0	R
Vancomycin	10	I
Ofloxacin	12	I
Chloramphenicol	15	I
Erythromycin	13	I
Penicillin	46	S
Cefoperazone	26	S
Ampicillin	26	S
Clindamycin	21	S

S stands for extremely sensitive; I stands for moderately sensitive; R stands for insensitive.

### 3.4 Minimum inhibitory concentration determination

**Figure 2:**
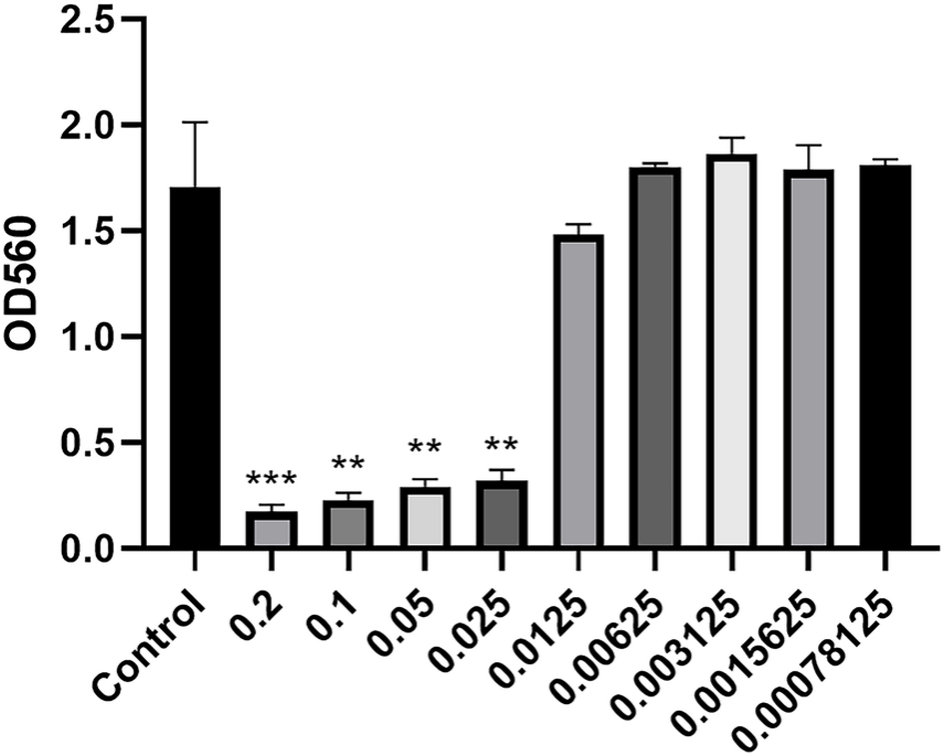
The minimum inhibitory concentration of Ea73 CFS against S. agalactiae.* p < 0.05; *** *p* < 0.001; **** *p* < 0.000 compared to control.

After 24 h of *S. agalactiae* culture, Ea73 CFS concentrations of 0.00625 g/mL, 0.003125 g/mL, 0.0015625 g/mL, and 0.00078125 g/mL had minimal effects on *S. agalactiae* proliferation, with no significant differences compared to the control group (0 mg/mL). However, Ea73 CFS concentrations of 0.2 g/mL, 0.1 g/mL, 0.05 g/mL, 0.025 g/mL, and 0.0125 g/mL significantly decreased *S. agalactiae* proliferation compared to the control group (Figure 2).

### 3.5 Effect of Ea73 CFS on *S. agalactiae* biofilm

#### 3.5.1 Film formation ability assay

**Figure 3:**
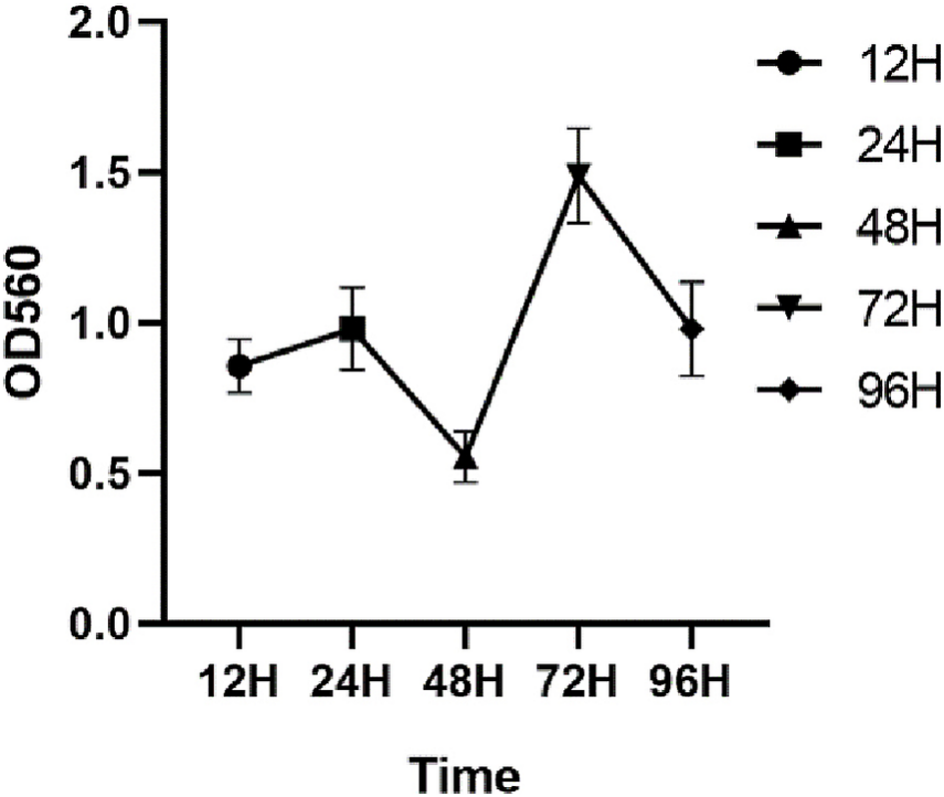
Biofilm formation ability of *S. agalactiae.*

As shown in Figure 3, the OD values increased progressively with an increase in the incubation time, attaining a peak value of 1.01 at 24 h, indicative of a robust biofilm-forming capacity in this strain (21), and the OD peak suggests that the biofilm at this time reached the maturation stage. The OD value decreased at 42 h, and this stage corresponded to the dispersion stage of the biofilm, where part of the biofilm detached from the bacteria and returned to the planktonic state, dispersed to new parts of the bacteria, and carried out a new round of biofilm colonization at this point of disintegration and dispersal, which corresponded to the secondary elevation of the OD value at 72 h.

#### 3.5.2 Laser confocal microscopy results

**Figure 4:**
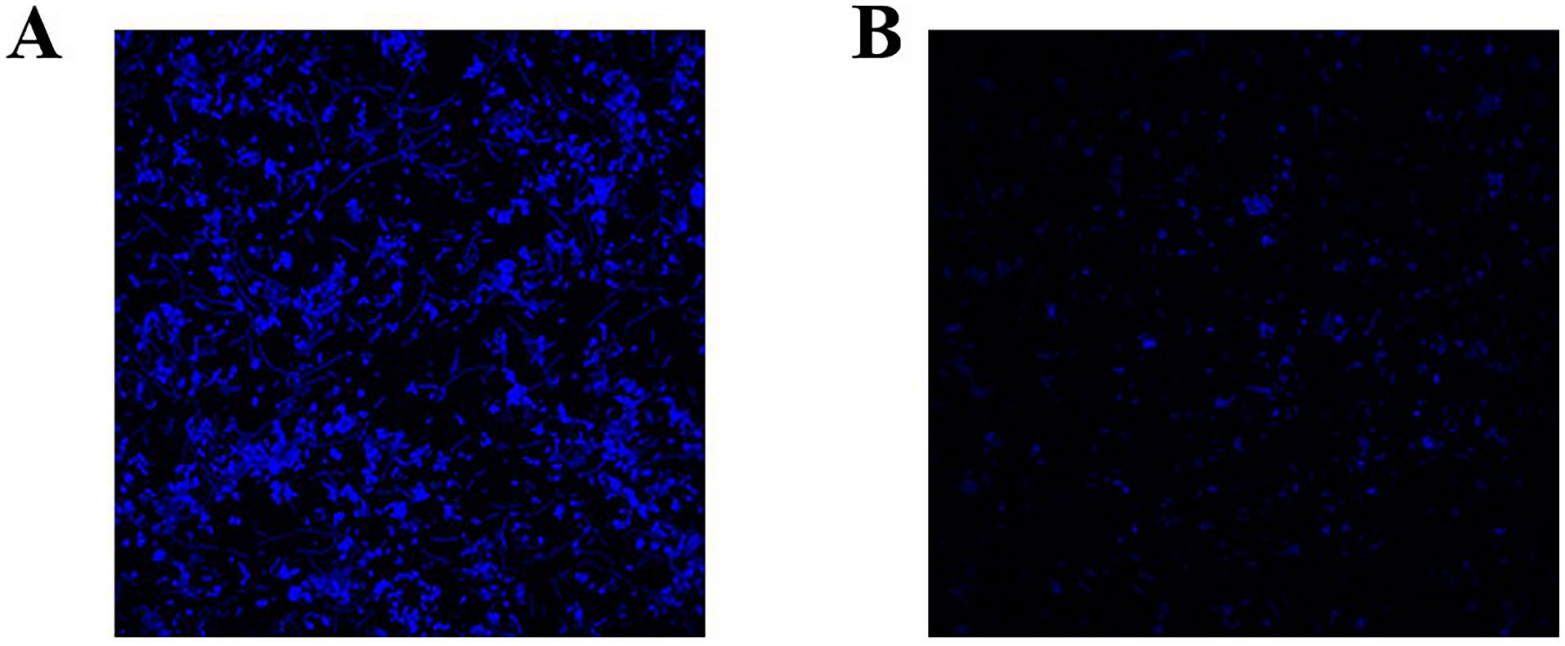
Representative *S. agalactiae* biofilms stained with LIVE/DEAD Kit and analyzed using fluorescence microscopy. **(A)** Control group and **(B)** 1/2 MIC group. The purple fluorescence indicates the live cells, whereas the red fluorescence indicates the dead cells or cells with a damaged cell wall.

Microscopic observations revealed that after treatment with 1/2 MBIC (5 mg/mL) of Ea73 CFS, the fluorescence intensity of the *S. agalactiae* standard strain decreased significantly (Figure 4). The three-dimensional structure of the biofilm disintegrated, with most of the biofilm becoming loose and scattered. These results indicate that Ea73 CFS treatment disrupts the three-dimensional structure of the *S. agalactiae* biofilm, reducing bacterial adhesion and aggregation, leading to the excretion of intracellular substances and bacterial death.

#### 3.5.3 Effect of Ea73 CFS on bacterial metabolism in *S. agalactiae* biofilms

**Figure 5:**
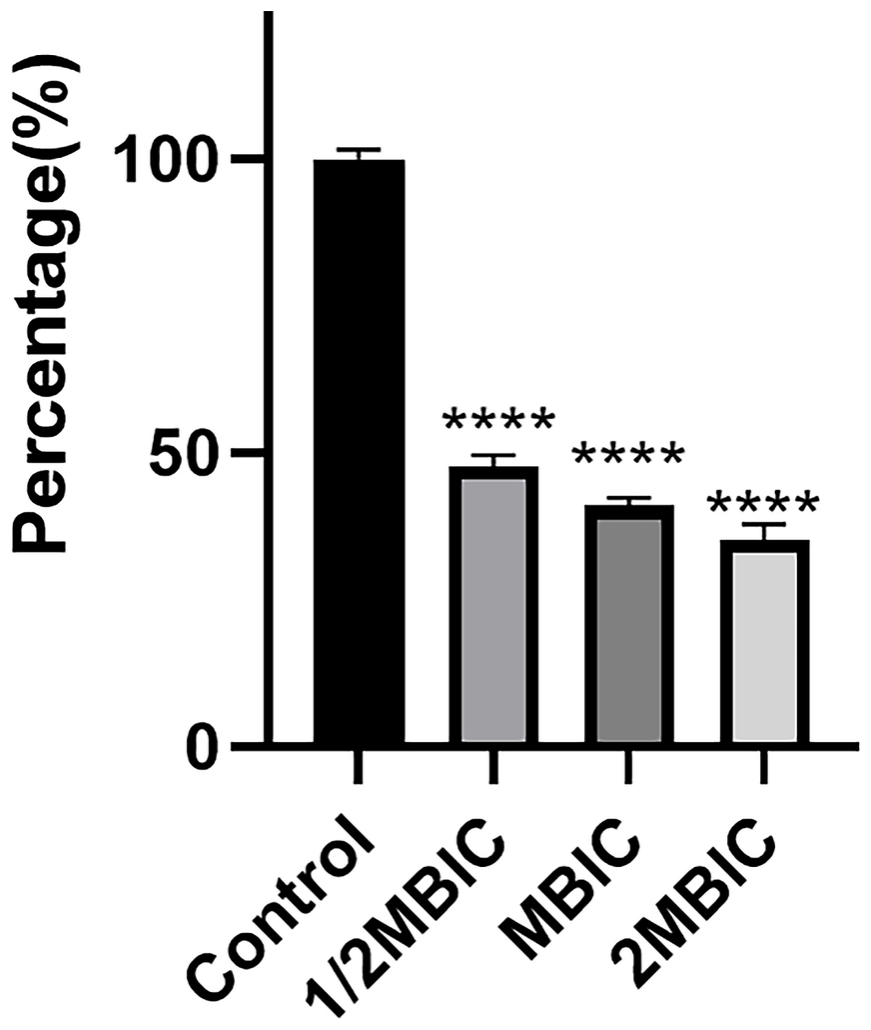
Effects of Ea73 CFS on bacterial metabolism in *S. agalactiae* biofilms.* *p* < 0.05; *** *p* < 0.001; **** *p* < 0.000 compared to control.

The metabolic capacity of bacteria can, to a certain extent, reflect the viability of bacterial cells. As shown in Figure 5, after treatment with Ea73 CFS at concentrations of 1/2MBIC, MBIC, and 2MBIC, the metabolic activity of the *S. agalactiae* biofilm decreased by 52.2, 58.9, and 64.8%, respectively. This may be because the electron transport chain for energy transfer during bacterial metabolism is mainly located on the inner surface of the cell membrane. However, after being stimulated and damaged by Ea73 CFS, the cell membrane loses its function of transferring energy and conducting metabolism, and thus its metabolic activity is greatly reduced.

#### 3.5.4 Enzyme hydrolysis experiment

**Figure 6:**
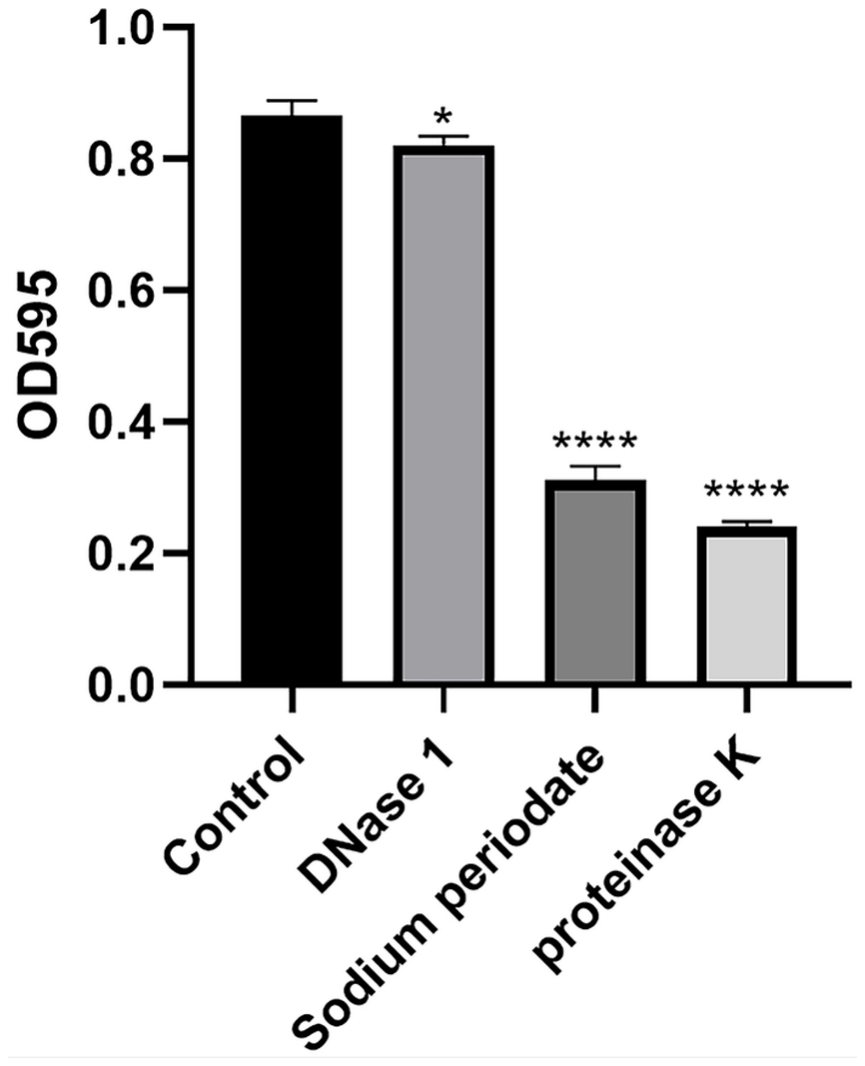
Enzymatic hydrolysis experiment. * *p* < 0.05; *** *p* < 0.001; **** *p* < 0.0001 compared to control.

As shown in Figure 6, the amount of biofilm decreased the most after proteinase K treatment, by approximately 72.2%, while the amount of biofilm decreased the least after DNase I treatment, by only 5.2%. The results also showed that the highest content of *S. agalactiae* biofilms was protein, followed by exopolysaccharides and eDNA.

#### 3.5.5 Effect of Ea73 CFS on extracellular proteins

**Figure 7:**
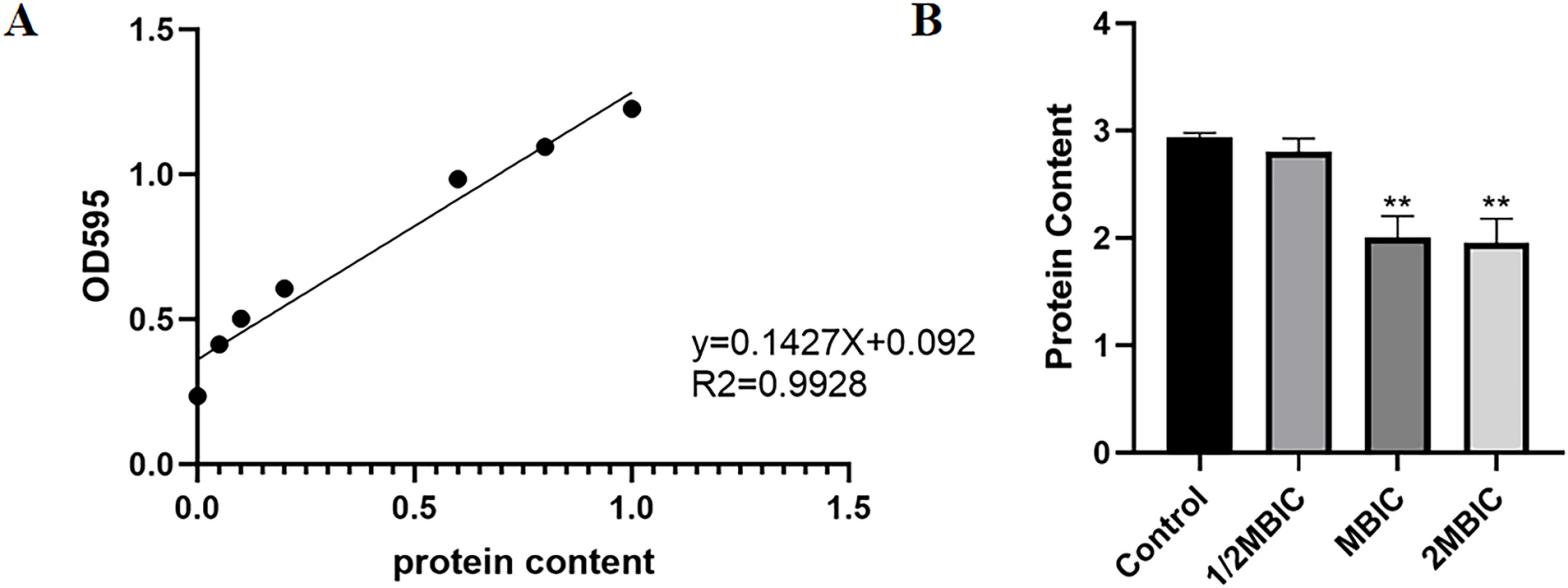
**(A)** Standard curve of protein; **(B)** Effect of Ea73 CFS on extracellular proteins in *S. agalactiae* biofilms.* *p* < 0.05; ** *p* < 0.01 compared to control.

As shown in Figure 7A, the standard curve equation for protein is: y = 0.1427X + 0.092 (*R*^2^ = 0.9928). Using this standard curve, the effect of Ea73 CFS on the extracellular protein content in *S. agalactiae* biofilms were determined. As shown in Figure 7B, treatment with 20 mg/mL of Ea73 CFS reduced the extracellular protein content by 87.92%. This indicates that Ea73 CFS significantly inhibits the formation of extracellular proteins in *S. agalactiae* biofilms and significantly suppresses their synthesis and secretion.

### 3.6 Ea73 CFS network pharmacology analysis results

#### 3.6.1 Common targets of the main components of Ea73 CFS and *S. agalactiae*-mediated diseases

**Figure 8:**
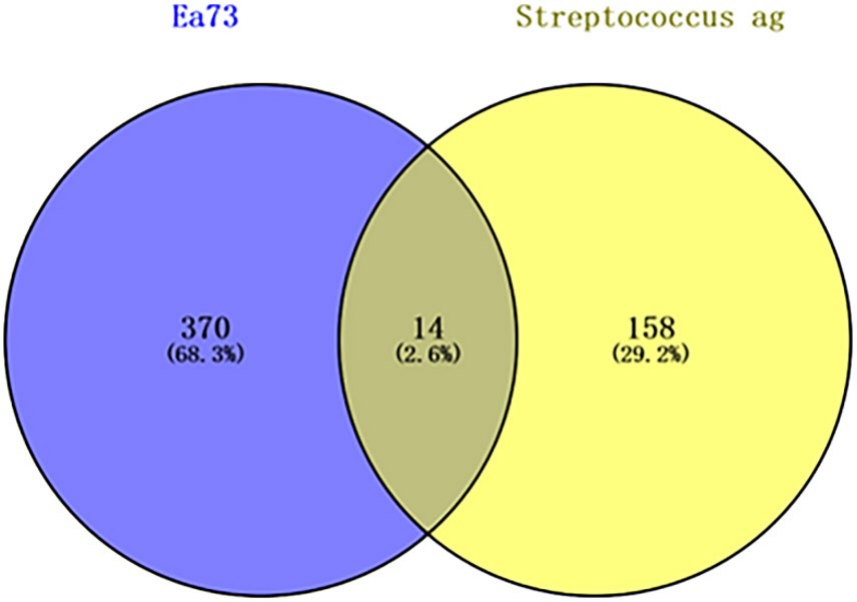
Venn diagram of Ea73 CFS treatment targets for *S. agalactiae*-mediated diseases.

A Swiss Target Prediction analysis (*p* > 0.5) identified 370 potential active ingredient targets of EA73 CFS and 158 *S. agalactiae*-mediated disease targets through the GeneCards and DisGeNET databases. These targets were imported into Venn 2.1.0 and the intersection of the two databases was determined, resulting in 14 shared targets, as shown in the Figure 8.

#### 3.6.2 Ea73 CFS main component-target-GBS-mediated disease network

**Figure 9:**
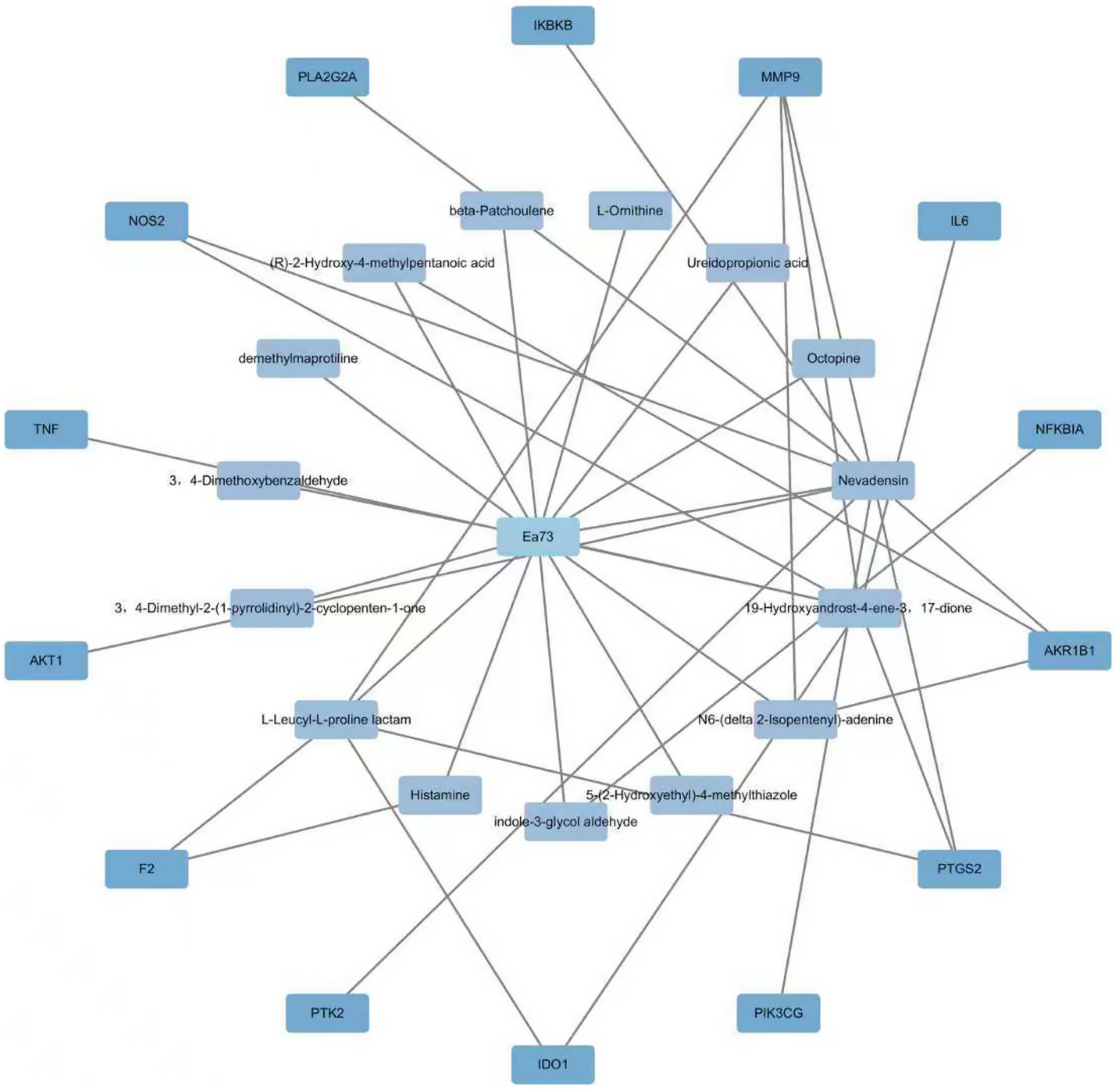
Ea73 CFS treatment of *S. agalactiae*-mediated disease target network diagram.

The Swiss Target Prediction database identified 14 potential targets for Ea73 CFS main components. These targets were imported into Cytoscape 3.9.1 to create a visual network diagram, as shown in Figure 9. The connected edges represent the corresponding drug-active ingredient-target-disease interactions. Nevadensin, 19-Hydroxyandrost-4-ene-3,17-dione, and L-Leucyl-L-proline lactam had the most targets, with degree values of 15, 10, and 5, respectively, suggesting that these components may play an important role in interactions with *S. agalactiae*-mediated diseases. At least two active ingredients were linked to each of the protein targets, including MMP9, PTGS2, AKR1B1, NOS2, IDO1, and F2. This suggests that these protein targets may be potential targets for *S. agalactiae*-mediated diseases.

#### 3.6.3 PPI network

**Figure 10:**
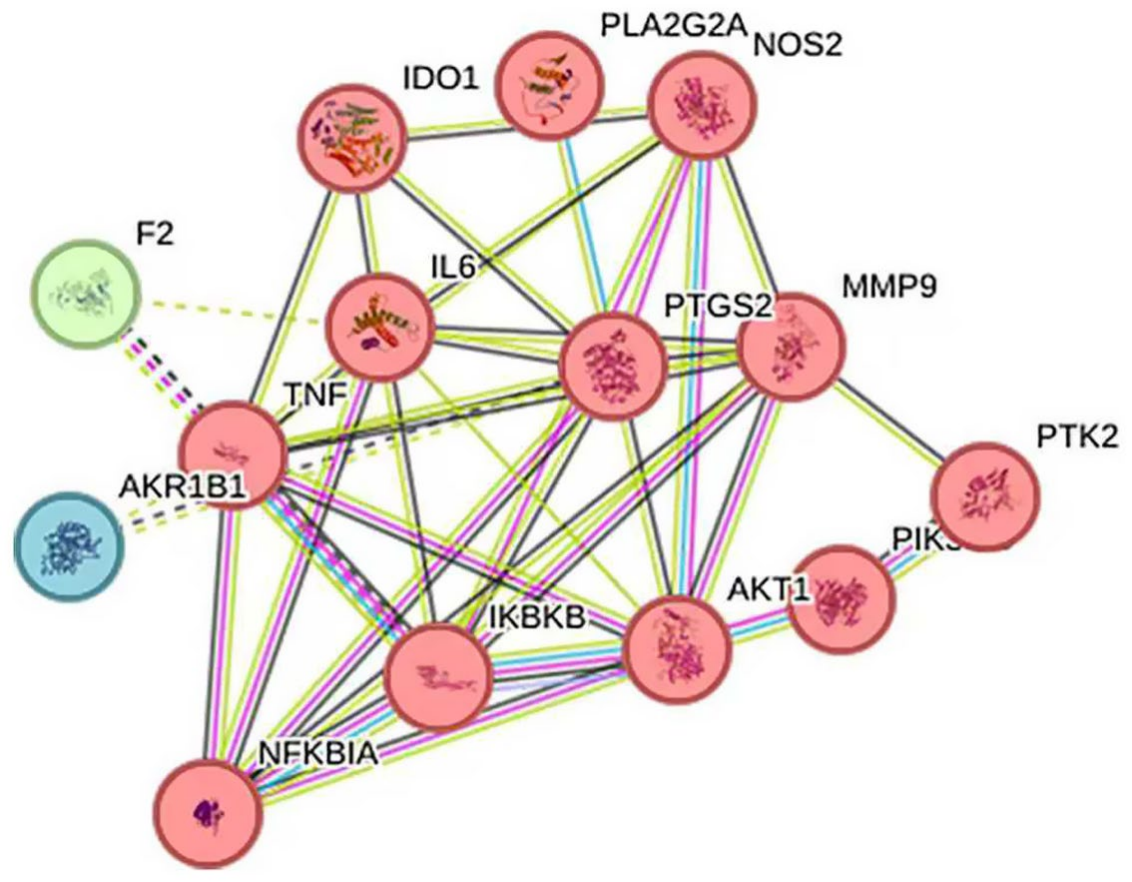
PPI network of Ea73 CFS-related targets.

The 14 intersection targets obtained in 2.6.1 were input into the String database, and the species “*Streptococcus agalactiae*” was selected to construct a protein–protein interaction network (Figure 10). This network had 14 nodes, 10 edges, and an average degree of 5.71. Protein targets with high degrees in this network include TNF, PTGS2, and IL6, with degrees of 10, 10, and 9, respectively. These targets may be important targets for Ea73 CFS in the treatment of bovine mastitis.

#### 3.6.4 GO enrichment analysis and KEGG pathway enrichment analysis

**Figure 11:**
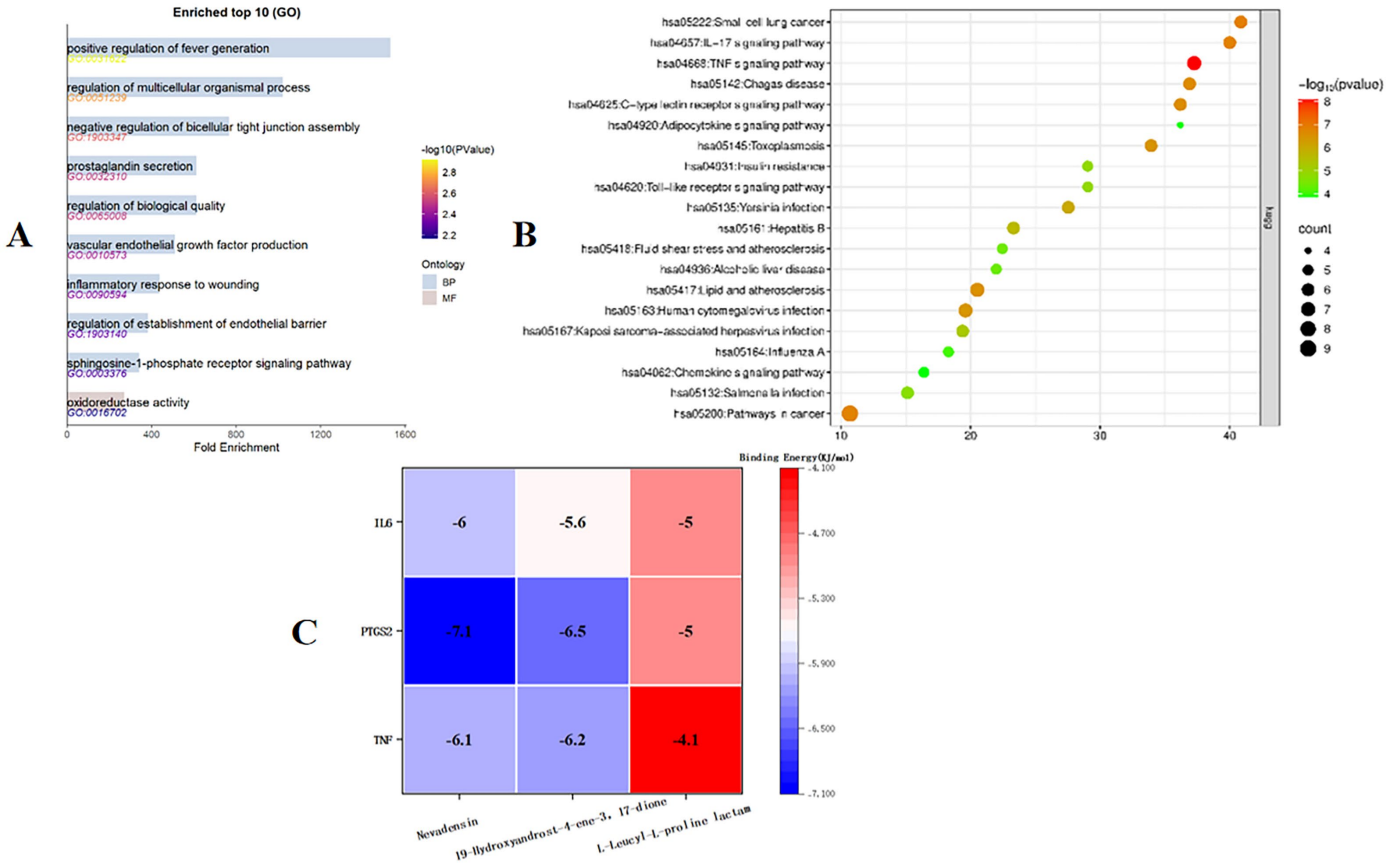
**(A)** Target GO enrichment analysis histogram. **(B)** Target signaling pathway enrichment analysis bubble chart. **(C)** Molecular docking binding energy heat map.

GO and KEGG enrichment analysis of the 14 targets identified above was performed using the David online database, and the results were visualized using the Microbiology Information online mapping website. A total of 387 GO enrichment analysis results were obtained. The top 10 fold enrichment results (*p* < 0.05) are shown in Figure 11, including 9 biological processes (BPs) and 1 molecular function (MF). BPs were primarily associated with positive regulation of angiogenesis, regulation of multicellular biological processes, negative regulation of tight junction assembly, and prostaglandin secretion; MFs were primarily associated with oxidoreductase activity.

KEGG pathway enrichment analysis, shown in Figure 11, yielded 20 pathways. The enrichment results suggest that the antimicrobial mechanism of Ea73 CFS may be closely related to pathways such as small cell lung cancer, the IL-17 signaling pathway, the TNF pathway, and the C-type lectin receptor signaling pathway.

#### 3.6.5 Molecular docking analysis

Autodock-vina was used to perform semi-flexible docking of the LC/MS–MS detection components and key targets. The resulting binding energy heat map is shown in Figure 11. The binding energy of the active ingredient of Ea73 CFS with the key target protein is basically lower than −5.0 kcal/mol, indicating that the receptor and ligand bind well. Among them, TNF, PTGS2, and IL6 have strong affinities with Nevadensin and 19-Hydroxyandrost-4-ene-3,17-dione, and hydrogen bonds are formed between the ligand and the protein, indicating that the structure after binding is in a highly stable state.

**Figure 12:**
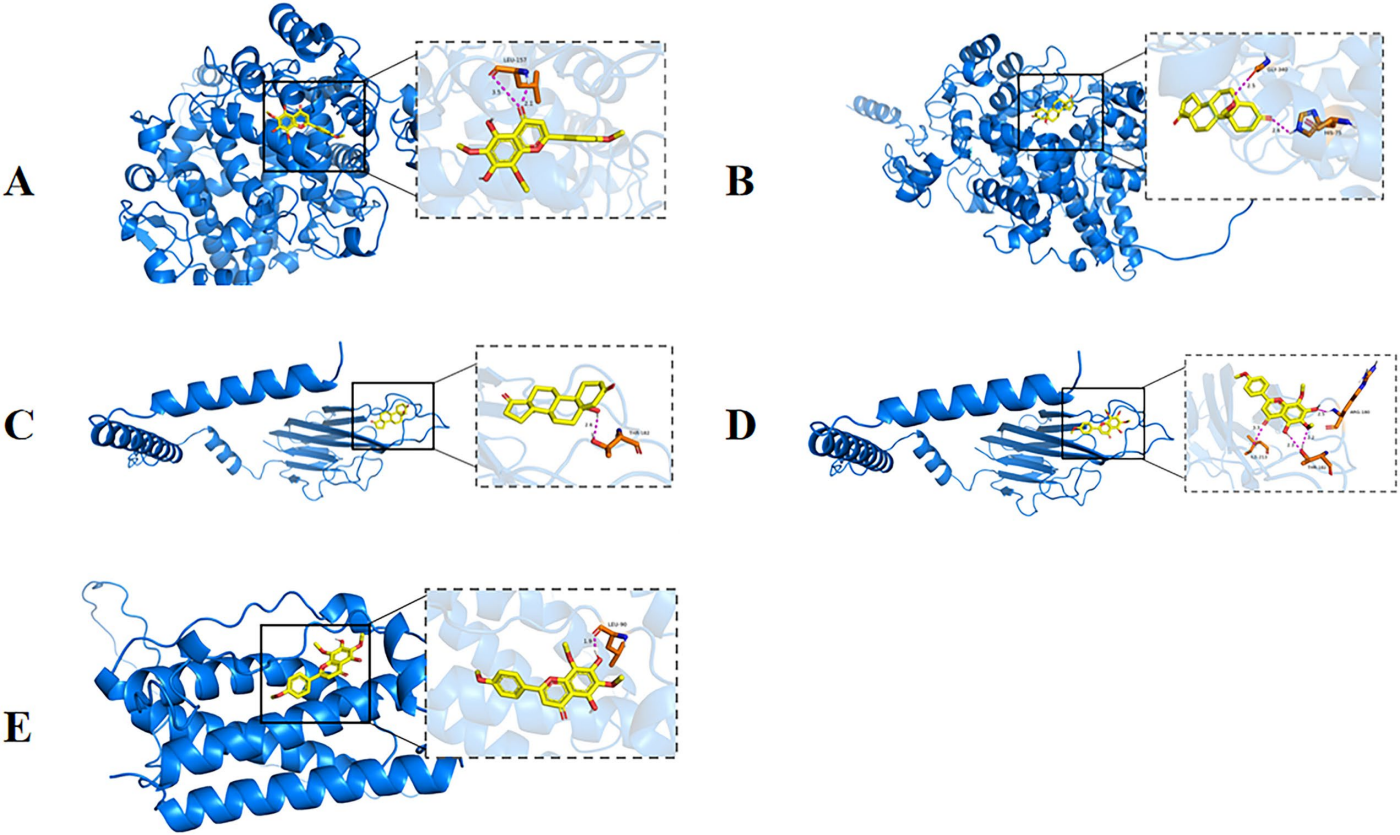
Partial diagram of molecular docking; **(A)** PTGS2-Nevadensin; **(B)** PTGS2-19-Hydroxyandrost-4-ene-3,17-dione; **(C)** AKT1-N6-(delta2-Isopentenyl)-adenine; **(D)** AKT1- Nevadensin; **(E)** IL6-Nevadensin.

As shown in Figure 12A, nevadensin a ligand that effectively binds to the active pocket of the PTGS2 protein has a binding energy of −7.1 kcal/mol, indicating relatively strong binding between the two. Three-dimensional analysis revealed that the ligand forms hydrogen bonds with the LEU-157 amino acid residue in the PTGS2 protein and hydrophobic interactions with the ARGA442, LYSA445, and PROA148 amino acid residues. These interactions facilitate nevadensin’s binding to the active pocket of the PTGS2 protein, leading to the formation of a complex. As shown in Figure 12B, 19-Hydroxyandrost-4-ene-3,17-dione a ligand that effectively binds to the active pocket of the PTGS2 protein has a binding energy of −6.5 kcal/mol, indicating relatively strong binding between the two. Three-dimensional analysis revealed that the compound forms hydrogen bonds with the GLY340 and HIS75 amino acid residues of PTGS2, promoting the binding of 19-Hydroxyandrost-4-ene-3,17-dione to the active pocket of the PTGS2 protein, leading to the formation of a complex. As shown in Figure 12C, 19-Hydroxyandrost-4-ene-3,17-dione, as a ligand, binds effectively to the active pocket of the TNF protein with a binding energy of −6.2 kcal/mol, indicating relatively strong binding. Three-dimensional analysis revealed that the ligand forms hydrogen bonds with amino acid residue THR182 of the TNF protein and hydrophobic interactions with residues TRPA191, CYSA146, and LYSA189. These interactions facilitate the binding of 19-Hydroxyandrost-4-ene-3,17-dione to the active pocket of the TNF protein, ultimately forming a complex. As shown in Figure 12D, nevadensin, as a ligand, effectively binds to the active pocket of the TNF protein with a binding energy of −6.1 kcal/mol, indicating relatively strong binding between the two. Three-dimensional analysis revealed that the ligand forms hydrogen bonds with amino acid residues ILE213, THR182, and ARG180 of the TNF protein, and hydrophobic interactions with amino acid residues GLUA193 and CYSA146. These interactions facilitate the binding of nevadensin to the active pocket of the TNF protein, leading to the formation of a complex. As shown in Figure 12E, nevadensin, as a ligand, effectively binds to the active site of the IL6 protein with a binding energy of −6.0 kcal/mol, indicating relatively strong binding between the two. Three-dimensional analysis revealed that the ligand forms hydrogen bonds with the LEU90 amino acid residue of the protein and hydrophobic interactions with the LYSA94 and LEUA189 amino acid residues. These interactions facilitate nevadensin’s successful binding to the active site of the IL6 protein, ultimately forming a complex.

## 4 Discussion

This study determined the minimum inhibitory concentration of Ea73 CFS against *S. agalactiae* and found that when the concentration of Ea73 CFS was 12.5 mg/mL, *S. agalactiae* showed significant inhibition. This was consistent with our previous study in that showed that at the same concentration, the inhibition diameter of Ea73 extract against *Escherichia coli* ATCC 35218, *Salmonella* H9812 and *Staphylococcus aureus* were approximately 10.57 ± 0.18 mm, 9.69 ± 0.17 mm, and 32.16 ± 2.04 mm, respectively, (9).

*S. agalactiae* has the characteristic of biofilm formation, which can effectively evade the immune clearance, phagocytosis, and bactericidal effects of traditional antibiotics in the body. Therefore, the biofilm of *S. agalactiae* plays an important role in causing subclinical mastitis and recurrent infections in dairy cows (2). Once pathogens form biofilms, they can effectively resist the entry of antibiotics into the membrane, reduce bacterial sensitivity to antibiotics, increase treatment difficulty, prolong medication time, and result in poor clinical treatment outcomes (22, 23). Bacteria can form small colony mutants within the biofilm in response to strong immune responses from infected hosts and induction by antibiotics, thus remaining in the host for a long time (24, 54). In this study, it was determined that the biofilm formation condition of the strain was at its peak after culturing at 37 °C for 24 h, indicating that the biofilm was in the mature stage at this time. In contrast, various studies have reported peak biofilm formation at 48 h incubation (25). The difference in the outcomes from our study may be due to the difference in culture media used in our studies (26). The minimum membrane inhibitory concentration of Ea73 CFS on *S. agalactiae* was determined to be 10 mg/mL according to the plate coating method. Laser confocal microscopy further confirms that treatment with 1/2MBIC of Ea73 CFS destroyed 90% of the biofilm stereo structure of *S. agalactiae*, leading to bacterial death and reducing bacterial population. This results are in consistent with results for other studies that show that the cell-free supernatant of probiotic bacteria such as *Lactobacillus* and *Bacillus* spp. exerted antibiofilm and antibacterial activities against various pathogenic bacteria (27–29).

Furthermore we observed that the metabolic activity of *S. agalactiae* biofilm was significantly reduced after treatment with Ea73 CFS, and the degree of reduction increased with increasing concentration. Similar results were observed in the study by Islam et al. (28) which reported that cell-free supernatants (CFSs) from the Culture of *Bacillus subtilis* inhibited *Pseudomonas* sp. biofilm formation.

One essential element and fundamental building block of the three-dimensional structure of bacterial biofilms is EPS (30). As can be seen in Figure 6, the extracellular polysaccharides and extracellular proteins of the *S. agalactiae* EPS matrix were reduced by EA73 CFS, and the main structure of the *S. agalactiae* EPS matrix was disrupted. This finding is consistent with results that report that some *Bacillus* CFSs have a disruptive effect on the three-dimensional structure of *S. aureus* biofilms (16, 31). Further studies on the extracellular protein content showed that the proteins of *S. agalactiae* biofilm decreased by 87.92% when the concentration of Ea73 CFS was 20 mg/mL. Therefore, we speculated that Ea73 CFS can clear the biofilm and accelerate the death of *S. agalactiae* by inhibiting the synthesis and secretion of the protein component of the EPS.

From the GO results, the positive regulation of angiogenesis from the BP results suggests that Ea73 CFS downregulates angiogenesis related signaling pathways (such as the VEGF pathway). Reducing angiogenesis at the site of infection limits the spread and nutrient supply of *S. agalactiae* (32). EA73 CFS may regulate the activation of multicellular biological processes, which reshape the infectious microenvironment and synergistically inhibit the pathogenicity of *S. agalactiae* by regulating the migration of inflammatory factors (such as IL-6, TNF - *α*) and immune cells (such as macrophages and neutrophils). Moreover, we also observed a negative regulation of tight junction assembly in cells. This finding implies that Ea73 CFS may enhance cell permeability and promote immune cell infiltration to clear pathogens by disrupting tight junction proteins (such as ZO-1, occludin) in breast epithelium (33). Prostaglandin secretion is directly related to PTGS2 function (34). Combined with the docking results, Nevadensin inhibits PTGS2 activity, suggesting that Ea73 CFS may alleviate local inflammation by inhibiting the synthesis of inflammatory mediator prostaglandin E2, reducing the impact on vascular permeability and pain response (35). The regulation of oxidoreductase activity may be achieved by modulating the redox balance of the host pathogen microenvironment, such as enhancing the activity of antioxidant enzymes such as superoxide dismutase (SOD) to alleviate oxidative stress damage, thereby inhibiting the survival of *S. agalactiae* (36). This therefore indicates that Ea73 CFS inactivates *S. agalactiae* by targeting/inhibiting oxidoreductases (such as thioredoxin reductase) hence further weakening its pathogenicity.

From the KEGG pathway, we observed the association of Ea73 CFS withsmall cell lung cancer-related pathways. Thus, this suggests that Ea73 CFS may inhibit epithelial mesenchymal transition (EMT), maintain the integrity of the breast epithelial barrier, and reduce the opportunity for pathogen invasion. The negative regulation of IL-17 pathway by Ea73 CFS indicates that Ea73 can reduce neutrophil infiltration and release of pro-inflammatory cytokines (such as IL-1β, IL-6), thereby alleviating excessive inflammatory response caused by *Streptococcus agalactiae* infection and avoiding tissue damage. In addition, the association between the IL-17 pathway and Th17 cell differentiation may be inhibited by endophytic bacteria by regulating the balance of Treg cells, further stabilizing the immune microenvironment (37). The regulation of TNF pathway by Ea73 (such as downregulating TNF-*α* and TNFR1 expression) indicates that Ea73 can block the activation of NF-κB and MAPK signaling pathways, and inhibit the inflammatory cytokine storm induced by *S. agalactiae* (such as IL-8 and MCP-1). Furthermore, due to the association between TNF pathway and cell apoptosis, Ea73 CFS may indirectly activate anti apoptotic signals (such as PI3K/Akt) to protect host cells from pathogen toxin damage (38, 39).

Molecular docking showed that Nevadensin and 19-Hydroxyandrost-4-ene-3, 17 dione components can effectively bind to TNF, PTGS2, and IL6 protein receptors. TNF, PTGS2, and IL6 are all host proteins. TNF-α (tumor necrosis factor alpha) is a core inflammatory mediator in *S. agalactiae* infection (40). Numerous researches have shown that *S. agalactiae* activates the TNF-α signaling pathway in the host immune system, triggering a strong inflammatory response and promoting the recruitment and activation of neutrophils and macrophages, thereby enhancing its ability to clear bacteria (40–43). In addition, TNF-α can further amplify the inflammatory cascade by activating the NF-κB and MAPK signaling pathways, leading to tissue damage and pathological changes (44, 45). PTGS2 (prostaglandin endoperoxide synthase 2, also known as COX-2) is mainly involved in the synthesis of inflammatory mediators in *S. agalactiae* infection (46). This enzyme catalyzes the conversion of arachidonic acid into prostaglandin E2 (PGE2), which exacerbates local inflammation by regulating vascular permeability and pain response (47). Studies have reported that host tissues without *Streptococcus* spp. infection, overexpression of PTGS2 is closely associated with inflammatory tissue damage, and its activity is positively regulated by the NF-κB and IL-1β signaling pathways [(48); LaRock and Nizet, 2014]. IL-6 (interleukin-6) is a key immune regulatory factor in *Streptococcus agalactiae* infection (49). It promotes Th17 cell differentiation by activating the JAK-STAT3 signaling pathway, enhances neutrophil mediated bacterial clearance ability, but may also trigger excessive inflammatory responses (such as cytokine storms) (50, 51).

The major limitation of the study is not identifying specific compounds from the many secondary metabolites produced by this endophyte and investigating its antibacterial properties. Thus, in future studies, the plan is to identify and isolate anti-biofilm-specific secondary metabolites and confirm their activity on various pathogenic bacteria.

## 5 Conclusion

This study demonstrates that *Bacillus velezensis* Ea73 CFS can effectively inhibit the proliferation of bovine-derived *S. agalactiae* via multi-target pathways including inhibiting biofilm formation, regulating inflammatory factors, and immune function.

Therefore, bioactive compounds with anti-biofilm activities can be isolated from *Bacillus velezensis* EA73. These results provide a theoretical basis for the development of new antibacteria agents against *S. agalactiae* and other biofilm forming bacteria.

## Data Availability

The datasets presented in this study can be found in online repositories. The names of the repository/repositories and accession number(s) can be found in the article/supplementary material.

## Footnotes

^1^https://pubchem.ncbi.nlm.nih.gov/, accessed on 12 April 2025

^2^ http://www.swisstargetprediction.ch/

^3^https://www.disgenet.com/, accessed on 12 April 2025

^4^ https://www.Genecards.org

^5^https://bioinfogp.cnb.csic.es/tools/venny/, accessed 15 April, 2025

^6^https://string-db.org/, accessed on 15 April, 2025

^7^https://david.ncifcrf.gov/, accessed on 12 April, 2025

^8^https://www.bioinformatics.com.cn/, accessed on 12 May, 2025

^9^ https://pubchem.ncbi.nlm.nih.gov/
